# Sessile multidroplets and salt droplets under high tangential electric fields

**DOI:** 10.1038/srep25002

**Published:** 2016-04-28

**Authors:** Guoxin Xie, Feng He, Xiang Liu, Lina Si, Dan Guo

**Affiliations:** 1 State Key Laboratory of Tribology, Tsinghua University, Beijing 100084, China; 2 School of Mechanical Engineering, Beijing Institute of Technology, Beijing 100081, China

## Abstract

Understanding the interaction behaviors between sessile droplets under imposed high voltages is very important in many practical situations, e.g., microfluidic devices and the degradation/aging problems of outdoor high-power applications. In the present work, the droplet coalescence, the discharge activity and the surface thermal distribution response between sessile multidroplets and chloride salt droplets under high tangential electric fields have been investigated with infrared thermography, high-speed photography and pulse current measurement. Obvious polarity effects on the discharge path direction and the temperature change in the droplets in the initial stage after discharge initiation were observed due to the anodic dissolution of metal ions from the electrode. In the case of sessile aligned multidroplets, the discharge path direction could affect the location of initial droplet coalescence. The smaller unmerged droplet would be drained into the merged large droplet as a result from the pressure difference inside the droplets rather than the asymmetric temperature change due to discharge. The discharge inception voltages and the temperature variations for two salt droplets closely correlated with the ionization degree of the salt, as well as the interfacial electrochemical reactions near the electrodes. Mechanisms of these observed phenomena were discussed.

The dynamics and stability of liquid droplets subjected to a high electric field are of immense scientific interest and of key importance to many applications, e.g., oil recovery technologies^1^, lab-on-a-chip devices^2^, applications relevant to wetting/spreading^3^,^4^,^5^. In recent years, a significant body of research works has been conducted on the droplet dynamics on a solid surface (i.e., sessile droplet) under the influence of an imposed electric field. The wetting and spreading behaviors of droplets can be tuned by applying electric fields perpendicular to the solid surface, namely electrowetting/spreading behavior^3^,^4^,^5^,^6^,^7^,^8^,^9^,^10^,^11^.

On the other hand, the behaviors of water sessile droplets subjected to horizontal electric fields have attracted great attention. Water droplets on the insulator’s surfaces due to rain or fog condensation are a main factor to local intensification of the electric field, as well as the resulting occurrence of coalescence, discharge and flashover between droplets^12^,^13^. Similar to the droplet in another fluid, the sessile droplet firstly experiences deformation or vibration before the occurrence of coalescence and discharge/arc after applying an electric field^14^. A number of factors could affect the electric field enhancement as well as the resultant droplet deformation and discharge behavior^14^,^15^,^16^,^17^,^18^,^19^,^20^,^21^. The magnitude of discharge current, surface moisture-resistivity and surface hydrophobicity played the dominant roles in the low current discharge process^13^,^22^.

Different experimental techniques have been employed to study the deformation and discharge behaviors between water droplets on the insulator surface, including high-speed photography, pulse current measurement and their combination^13^,^20^,^21^,^23^, as well as RF radiation sensing^24^, ultrahigh frequency (UHF) technique^12^,^25^, and atomic emission spectroscopy^26^. The discharge temperatures between two water droplets have been measured with optical emission spectroscopy by Xiao, *et al*.^27^ and with infrared thermography by our group^28^.

Despite these progresses, the interaction processes of sessile multidroplets and salt droplets subjected to high tangential electric fields have been rarely discussed. The understanding on the dynamics of these droplets and the resulting discharge behaviors are not only of great scientific significance, but also relevant to the practical degradation issues of high-voltage insulators. In the present work, on the basis of infrared thermography, high-speed photography and pulse current measurement, the droplet coalescence and discharge activity between sessile multidroplets as well as salt droplets under high tangential electric fields, will be investigated. The schematic diagram of the experimental setup is shown in Fig. 1. Briefly, aqueous droplets were placed on flat horizontal hydrophobic insulator’s surfaces, and metal electrodes were dipped into the center point of the droplets. DC voltages between the electrodes were applied and increased gradually until the discharge inception between droplets. The side views of the temperature variation and the movement of droplets were obtained by using an infrared thermographic camera. Meanwhile, the top views of the droplet behavior were also visualized with an optical microscope.

## Results

### Two-droplet configuration

Thermographic pictures of the side-view of two water droplets on silicone rubber (SR) surfaces under high positive voltages are shown in Fig. 2. The left electrode was energized, and the right one was grounded. Positive case/discharge was defined as that when a positive voltage was applied onto the energized electrode, and vice versa. Moreover, Region *A* indicated with a dashed frame in Fig. 2 is the region where the maximum and average temperatures were measured. These definitions will be also applicable in the following part. In Fig. 2(a), the semi-spherical dark areas representing the water droplets (temperature: 31 °C), due to the slight temperature contrast as compared with the surrounding temperature (Δ*T* = −3 °C). The corresponding top-view bright-field optical image is shown in Fig. 2(m). Droplet deformation at the energized side, extending along the electric field direction towards the facing droplet (grounded droplet) could be seen when the voltage was increased to 5.5 kV [Fig. 2(b)].

When the voltage was increased further to 5.7 kV, a non-fixed discharge path could be seen with bright traces (indicated by Arrow 1, temperature: 35 °C), moving across the faces of the droplets, as shown in Fig. 2(c). The corresponding top-view bright-field optical image at this instant is shown in Fig. 2(n). Moreover, it can be observed that continuous discharge initiated from the energized droplet to the grounded one. The temperature rise of the left part of the grounded droplet took place (temperature: 36 °C), as shown in Fig. 2(d). The thermal field rapidly diffused to the right part of the grounded droplet, as shown in Fig. 2(e). After that, the temperature around the discharge root of the grounded droplet continued to increase, and meanwhile, the temperature of the energized droplet started to increase. At 150 ms, the temperature distribution of the droplet at the grounded droplet (maximum: 46 °C) was more uniform than the energized one (maximum: 42 °C), as shown in Fig. 2(f). Moreover, the temperature also increased in the part between droplets (maximum: 45 °C), suggesting that a thin layer of water from the droplets was established on the solid surface, as evidenced by the top-view bright-field optical image in Fig. 2(o), whereas the discharge path (average temperature: 36 °C) still moved across the droplet faces. Subsequently, the temperatures of both droplets increased dramatically, and generally asymmetric temperature distributions of the droplets (particularly the grounded droplet) could be seen, as shown in Fig. 2(g). Meanwhile, spread of both droplets with the reduction of the contact angle was also apparent, and a liquid bridge was then established between droplets, as evidenced by the top-view bright-field optical image in Fig. 2(p). Afterwards, the grounded droplet was drained gradually into the energized droplet, as shown in Fig. 2(h) where the temperatures of the liquid bridge and the constricted part of the grounded electrode were obviously higher (>60 °C). At this moment, the moving discharge path disappeared.

As time progressed, the grounded droplet detached from the electrode, resulting in intermittent discharge between the detached droplet and the grounded electrode [Fig. 2(i)]. The temperature of the left droplet was kept at around 60 °C, and evaporation caused the reduction of droplet size [Fig. 2(j)], giving rise to the droplet again detaching from the energized electrode [Fig. 2(k)]. Afterwards, intensive discharge between two electrodes and the intermediate merged droplet started. Finally, the liquid droplet between the electrodes tended to dry out, as shown in Fig. 2(l) as well as Fig. 2(r). After the negative voltage was applied onto the energized electrode and increased to the critical value (Fig. S1, Supplementary File), discharge started from the grounded droplet to the opposite droplet, and the direction of discharge path was just opposite to that under positive discharge in Fig. 2.

Figure 3 shows the variations of the electric currents and the corresponding average temperatures of the area as indicated by the square frame (*A*) in Fig. 2 over time after discharge initiation between two droplets. As shown in Fig. 3(a), as compared with negative discharge, the electric current for positive discharge was slightly higher in the same time period (1.2–4.0 s), being consistent with the literature’s result^29^, where it was further suggested the higher electric current lead to the more intensive surface heating, as verified by our work [Fig. 3(b)].

### Three-droplet configuration

Thermographic pictures of the side-view of three aligned water droplets under high voltages are shown in Fig. 4. It indicated that droplet deformation started firstly at the energized droplet under high voltages of both polarities [Fig. 4(a,b,h,i)]. When the electric field was sufficiently large, discharge occurred instantly between the three droplets. Similar to the two-droplet configuration, the deformation of the grounded droplet was also obvious under a negative voltage at the instant of discharge inception. Occasionally, the discharge between the droplets was too fast to be captured (<10 ms). However, it was certain that the inserted droplet coalesced with the grounded droplet in the very short period after discharge initiation both for positive and negative discharges [Fig. 4(c,j)].

Afterwards, discharge between the energized droplet and the grounded bigger droplet persisted. Similar to the two-droplet case, discharge direction was from the high potential droplet to the low potential one, and the temperature of the latter firstly increased [Fig. 4(d,k)]. The grounded droplet became consequently more voluminous than the energized one. Afterwards, the two droplets had the tendency to form a liquid bridge [Fig. 4 (e) and (l)], followed by the drainage of liquid from the energized droplet to the grounded one [Fig. 4(f,m)]. As time progressed, the grounded bigger droplet detached from the electrode since the droplet size reduced gradually due to evaporation, and eventually the detached droplet spread and dried out [Fig. 4(g,n)]. The corresponding electric current variation curves to Fig. 4 are shown in Fig. S2 (Supplementary File), also suggesting that positive discharge was more intensive, and the temperature changes correlated well with the changes of electric currents.

The position of the inserted droplet was changed to investigate its influence on the droplet coalescence and discharge activity. The results of three aligned water droplets with the inserted droplet near the energized droplet under high voltages are shown in Figs S3 and S4 (Supplementary File). Generally, the discharge path direction could affect the location of initial droplet coalescence. The smaller unmerged droplet would be drained into the merged large droplet

### Salt droplets

In order to investigate the effect of addition of salt on the droplet coalescence and discharge characteristics of sessile droplets, three groups of the salt droplets were divided. Specifically, droplets of the NaCl solutions with different salt concentrations ranging from 0 to 1 M, droplets of salt solutions of different monovalent cations (Li^+^, Na^+^ and K^+^) and cations of different valences (Ca^2+^ and Al^3+^) were investigated.

The discharge inception voltages of these salt droplets are shown in Fig. 5, and the thermographic pictures of the discharge processes are shown in Figs 6 and 7. It could be seen in the left part of Fig. 6 that the discharge inception voltage decreased with the NaCl concentration. Thermographic pictures of these droplets in Fig. 6 show the detailed morphological and temperature changes, and apparently the key trends during discharge process are very similar to those in Fig. 2. Figure 6 also shows the discharge process was relatively violent at the low salt concentration and becomes mild at the high concentration [Fig. 6(e1–e4)], giving rise to more obvious temperature change in the droplets with the lower concentration salt. As the salt concentration increases, it could be also observed that the discharge process lasted increasingly longer during the electrolysis stage and the droplets hardly evaporated completely.

The right part of Figs 5 and 7 reflect the influence of different salt cations on the droplet coalescence and discharge behaviors. The discharge inception voltage decreased with the increasing ionic radius of the cations (Li^+^, Na^+^ and K^+^) [Fig. 7(b1–b3)], while the discharge process became slow and mild with the increasing ionic radius. For the 0.1 M LiCl droplets, a high temperature zone could be seen from [Fig. 7(d1,g1)] while the high temperature zone of 0.1 M NaCl and 0.1 M KCl disappeared at the later stages [Fig. 7(f2–g2)] and [Fig. 7(e3–g3)]. For the droplets of CaCl_2_ and AlCl_3_, similar characteristics in the initial part of discharge process [Fig. 7(a4)–(d4),(a5)–(d5)] were present. However, different morphological and temperature variations in the later discharge process [Fig. 7(e4–f4,e5–f5)] could be seen. The temperature rises of the merged droplets of CaCl_2_ and AlCl_3_ were more obvious near the grounded electrode than the energized electrode, while in contrast the temperature rise was higher near the energized electrode in the salt droplets with monovalent cations. In the work, a KCl droplet was also placed as the inserted droplet, and the energized and grounded droplets were of deionized water, as shown in Figs S5 and S6 (Supplementary File). Morphologically, relatively stable liquid bridges between these droplets formed due to the small temperature changes in these droplets, especially at the later stages.

## Discussion

From the previous parts, it can be seen that the interaction of water droplets on the solid surface under a strong horizontal electric field generally experienced the following processes:

1. Droplet deformation due to the electric field enhancement (*E*_*e*_) tangential to the surface^30^. Normally, the maximum *E*_*e*_ is in the horizontal direction near the three-phase point. In equilibrium, a force balance exists at the three phase point of the static droplet without the application of an electric field^13^,


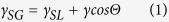


where *γ*_*SG*_, *γ*_*SL*_, *γ* and *Θ* are solid-gas interfacial tension and solid-liquid interfacial tension, liquid-gas interfacial tension, and Young’s angle, respectively. When an electric field is applied, a polarization stress would emerge for the energized droplet before discharge initiation^31^, resulting in the contact line deformation of droplet.

2. When *E*_*e*_ exceeds a critical value, the release of charges from the high potential droplet to the low potential droplet occurred. Presumably, positive charges near the tip of the deformed droplet, which also give their momentum to neutral gas molecules by collisions under the high electric field, drift to the facing grounded droplet under the electrostatic force^32^. Obvious polarity effect of the discharge path direction from the high potential droplet to the low potential droplet, and the larger electric current of positive discharge as well as the resultant higher temperature rise have been observed. The reasons responsible for these observations could be roughly understood^33^: the dissolution of metal ions from the electrode with the higher potential could occur for positive discharge, and these ions would result in higher surface conduction. In the case of negative discharge, positive ions would migrate from the grounded electrode, resulting in the charge release from the grounded droplet to the energized one.

3. Droplet deformation was obvious before discharge inception and temperature rise, while the contact angle, particularly the advancing angle, did not reduce dramatically, and instead increased slightly during the droplet deformation. However, the contact angles decreased apparently after discharge initiated and the temperature was increased. It may suggest that temperature change, instead of the accumulated charges on the droplet surface, played the dominant role in affecting the surface tension variation of the droplet. Theoretically, asymmetric temperature rise could induce the presence of surface tension gradient in the droplet, and the higher temperature at the edge of water droplet at the facing sides resulted in the lower surface tension. The relationship between the surface tension, *γ* and temperature, *T* can be described as^34^


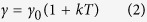


where *γ*_0_ is the surface tension at the critical temperature *T*_0,_ and *k* is the temperature coefficient of surface tension (*k* = −0.0015 °C for water).

In Fig. 2, the maximum temperature at the discharge root of the grounded droplet (*T*_n1_) immediately after discharge initiation [Fig. 2(d)] is ca. 36 °C (real temperature after calibration, *T*_r1_: 41.6 °C), and the corresponding water surface tension *γ*_1_ ≈ 71.3 mN/m. Besides the polarization force at the contact line, the capillary line force *F*_*c*_ in the horizontal direction due to temperature change in the droplet near the discharge root and the change of advancing angle *Θ*_*a*_ plays a very important role in the process of droplet elongation. The capillary line force *F*_*c*_ can be written as^35^,


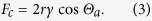


*F*_*c*_ acts on the contact line, perpendicularly to the contact line, and a positive value indicates the force points away from the droplet. The difference between the capillary line forces of the droplet near the discharge root between the instant without an electric field, and the instant when droplet elongation and movement occur after applying an electric field (Δ*F*_*c*_) is focused on. The analysis on the droplets in Fig. 2 will be taken as the example, and the temperature and the contact angle of the droplets have been measured. Then, we can get Δ*F*_*c-energied*_ = 0.112 mN for the energized droplet, and Δ*F*_*c-energied*_ = 0.117 mN for the grounded droplet, suggesting both droplets could protrude equally. It can be confirmed by the results in Fig. 2(g) where protrusions of close lengths could be seen.

The role of hydrostatic pressure variation in distorting the droplet shape is neglected because the Bond number 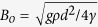 ≈ 0.33 < 1, where *g* and *ρ* are the acceleration of gravity and the liquid density. Then, the forces resisting droplet elongation and translation include the viscous force *F*_*v*_, the contact angle hysteresis *F*_*f*_, the contact line friction, *F*_*CLF*_ and the drag force from the electrode *F*_*d*_^9^,^36^. The former three forces are related with the droplet elongation and translation. The fourth force equates the frictional force of the contact line on the electrode, which is relevant to the droplet detachment. The viscous force *F*_*v*_ is^37^


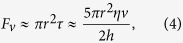


where *τ* is the shear stress, *η* is the liquid viscosity, and *v* is the motion velocity of the protrusion. The contact angle hysteresis *F*_*f*_ is^37^


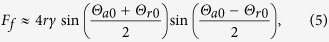


where *Θ*_*a*0_ and *Θ*_*r*0_ are the advancing and the receding contact angles, respectively. The variations of contact angles (advancing and receding angles) with and without an electric field are shown in Fig. S7 (Supplementary File).

The contact line friction *F*_*CLF*_ is roughly approximated as *F*_*CLF*_ ≈ *λv*^9^, where *λ* is the frictional factor (determined empirically). The drag force from the electrode *F*_*d*_ is 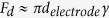
37.

Basically, protrusion from the droplet could be seen when the summation of polarization force and capillary force is larger than the contact angle hysteresis. When droplet movement starts before the droplet detaching from the electrode, the resistive forces include both the viscous force and the contact line friction. When the driving forces for the droplet movement are larger than the viscous force, the contact line friction and the drag force from the electrode *F*_*d*_, the droplet would detach from the electrode correspondingly.

For the grounded droplet in Fig. 2, *F*_*v*_ = ~10^−8^ N (*v* ≈ 6 mm/s for Fig. 2(f,g)), *F*_*v*_ ≈ 1.5 × 10^−8^ N, *F*_*CLF*_ = ~1.08 μN (*λ* is ~0.1), *F*_*d*_ ≈ 44 μN (*γ* is assumed to be constant). It can be inferred that *F*_*CLF*_ might be the main resistive force during the droplet elongation process while *F*_*d*_ might be the main force resisting the droplet detachment from the electrode.

Due to the changes of the electrocapillary line force, both droplets gradually spread accordingly. As time passed the protrusions joined together to form a liquid bridge, and ultimately the droplet detached from the electrode. Previously, it has been shown that the discharge was quite transient, followed by the formation of the liquid bridge between two droplets of a larger volume (3 μL) and a smaller separation (4 mm)^28^. More results on the relationship between the droplet coalescence/discharge and the droplet separation as well as the droplet size are shown in Fig. S8 (Supplementary File).

For the case of three aligned droplets, the above results showed that the direction of initial discharge path between three droplets was similar to the two-droplet cases. In our work, the dynamic behaviors of four aligned droplets with an unchanged separation between two extreme droplets exposed to high voltage were investigated. It was however difficult to get a reproducible result for determining the location where the coalescence of droplets firstly started because these droplets were quite close. Recent work also suggested that the evaporation rate difference could result in the asymmetric shape of droplets in very close proximity^38^, and this would add complexity to the droplet coalescence and discharge behaviors, especially for the three-droplet and four-droplet systems. Nevertheless, wherever droplet coalescence firstly started and whichever droplet was with the higher temperature, the unmerged droplet would be drained into the merged large droplet, primarily due to the fact that the pressure inside the smaller droplet was larger^39^.

The effects of salt concentration and type have been demonstrated to be important in the electro-coalescence behavior between droplets^40^,^41^,^42^. The discharge inception voltages in Fig. 5 suggested that discharge could be initiated between droplets more easily with the increasing salt concentration, the increasing ionic radius of the monovalent cations (Li^+^, Na^+^ and K^+^). As mentioned above, when the amount of ions is sufficiently large, discharge could occur near the deformed tip of the droplet due to the released charges colliding on the neutral gas molecules and then acting onto the facing droplet. It could be inferred that the inception of discharge closely related with the number of accumulated charges near the deformed tip. Apparently, the amount of charges could be increased with the increasing salt concentration.

In the case of droplets with different monovalent cations (Li^+^, Na^+^ and K^+^), the amount of charges at the deformed tip of the droplet correlated with the different degrees of ionization of the salts in the droplet, depending on the electronegativity difference between the cation and the anion of the salt compound. The electronegativity difference between K^+^ (0.82) and Cl^-^(3.16) was larger than those between Li^+^ (0.98)/Na^+^ (0.93) and Cl^−^(3.16)^42^, as verified by the electric conductivities of the salt solutions in Table 1. It could be expected that more ions dissolved in the droplet and accumulated near the deformed tip of the droplet under the influence of the electric field. Consequently, discharge inception voltage would be lowest for the KCl droplets and highest for the LiCl droplets, as shown in Fig. 5. In the case of CaCl_2_ and AlCl_3_ droplets, the electronegativity differences between Ca^2+^ (1.0)/Al^3+^ (1.61) and Cl^−^(3.16) were slightly smaller than those of K^+^ (0.82)/Na^+^ (0.93) and Cl^−^(3.16)^43^. As a result, the discharge initiations for CaCl_2_ and AlCl_3_ droplets were generally more difficult than those for NaCl and KCl droplets at the same salt concentration.

Furthermore, it has been mentioned above that for CaCl_2_ and AlCl_3_ droplets, the temperature rises of the merged droplets were more obvious near the grounded electrode than the energized electrode, which was contrary to the cases of the salt droplets with monovalent cations. For this phenomenon, a retrospect of the previous discussion on the temperature rise of the deionized water could tell us that the dissolution of metal ions from the electrode with the higher potential made a non-ignorable contribution to the higher surface conduction near the electrode with the lower potential, where less temperature rise will be expected.

Following this approach, the observed phenomenon could be understood as: Cations (Li^+^, Na^+^, K^+^, Ca^2+^ and Al^3+^, as well as H^+^ and dissolved Cu^2+^ from the anode) in the salt droplet would migrate from the energized droplet (anode) to the grounded droplet after a bridge was established between the two droplets, and in the same way, anions (Cl^−^ and OH^−^) would migrate towards the region adjacent to the energized electrode. Near the anode (energized electrode), chloride ions (Cl^−^) would donate electrons to the anode to form chlorine gas, i.e., 2Cl^−^(aqueous) -> Cl_2_(gas) + 2e-. Meanwhile, the hydroxyl ion (OH^−^) would also donate electrons to form water. In contrast, the electrochemical reactions were slightly different for the cations near the cathode (grounded electrode). In the cases of LiCl, NaCl and KCl droplets, hydrogen ions (H^+^) from water picked up electrons to form hydrogen gas, i.e., 2 H^+^ (aqueous) + 2e -> H_2_(gas). At the same time, the migrating copper ions (Cu^2+^) from the anode would also pick up electrons to form deposited metallic copper on the cathode. Under these circumstances, the part near the energized electrode (anode) would be more resistive than that near the grounded electrode in the merged LiCl, NaCl and KCl droplets due to the depletion of anions and the accumulation of residue cations (e.g., Li^+^, Na^+^, and K^+^) near the grounded electrode. In the cases of CaCl_2_ and AlCl_3_ droplets, the generation of Ca(OH)_2_ and Al(OH)_3_ (or AlO^2−^) would be possible, and then deposition of these chemical substances could increase the resistance of the merged droplet near the grounded electrode (cathode). Consequently, the temperature rises could be asymmetrically higher near the grounded electrode, being in contrast with the cases of LiCl, NaCl and KCl droplets, as evidenced by the results in Fig. 7.

In addition, the formation of morphologically stable liquid bridges between water droplets when the inserted droplet was conductive salt droplets was speculated as a result of the liquid bridge and the salt droplet being less resistive, giving rise to the small temperature rise and consequently the morphologically stable features.

## Conclusions

In the present work, infrared thermography, high-speed photography and pulse current measurement were used to investigate the droplet coalescence and discharge characteristics of sessile multidroplets and salt droplets under high DC tangential electric fields. For both two-droplet and three-droplet configurations, due to the anodic dissolution of metal ions from the electrode, the discharge path direction was from the high potential droplet to the low potential droplet at the initial stage after discharge initiation, and the temperature rise of the low potential droplet was more obvious. For the three-droplet configuration, the initial droplet coalescence between the inserted droplet and the extreme droplets were largely affected by the discharge path direction, and the small droplet would be drained into the merged large droplet due to the pressure difference in the droplets. The investigations on the effect of chloride salts on the droplet coalescence and discharge behaviors suggested that the discharge inception voltages between two salt droplets had been found to closely correlate with the electronegativity difference between the cation and the anion of the salt. The electrochemical reactions near the electrodes were important for the morphological and temperature variations in the salt droplets during the droplet coalescence and discharge processes.

## Methods

### Experimental setup

**Figure 1:**
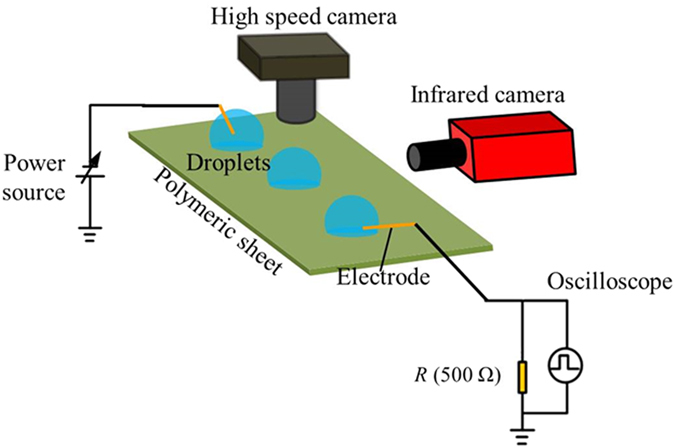
Schematic diagram of the experimental setup.

**Figure 2:**
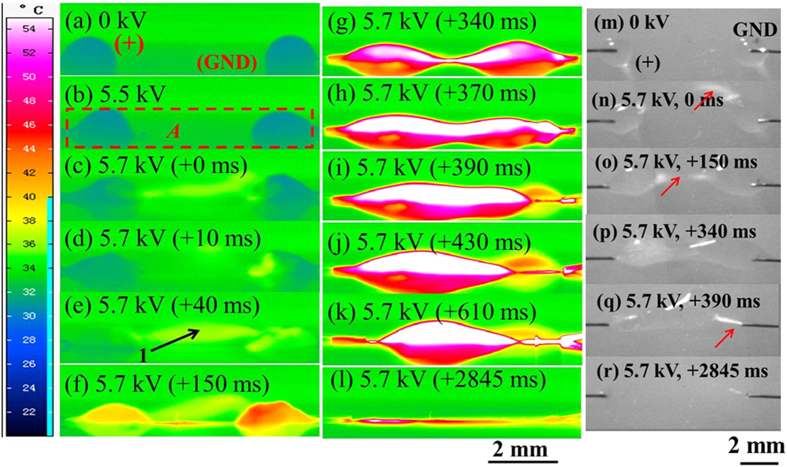
(**a–l**) Side-view thermographic pictures of two deionized water droplets (droplet volume: 2 μL, droplet separation: 7 mm) on the SR surface under a high positive voltage (the left electrode was energized, and the right one grounded); (**m–r**) Top-view bright-field optical images: (**m**) corresponds to (**a**); (**n**) corresponds to (**c**); (**o**) corresponds to (**f**); (**p**) corresponds to (**g**); (**q**) corresponds to (**i**); (**r**) corresponds to (**l**). The arrows indicate the discharge paths. The region *A* with a dashed frame in (**b**) is the region where the maximum and average temperatures were measured. The droplet volume was 2 μL, and the electrode separation was 7 mm. The droplet diameter (*d*) and height (*h*) were 1.79 mm and 0.98 mm, and the contact radius (*r*) between the droplet and the solid surface (i.e., droplet footprint radius) was 0.89 mm.

**Figure 3:**
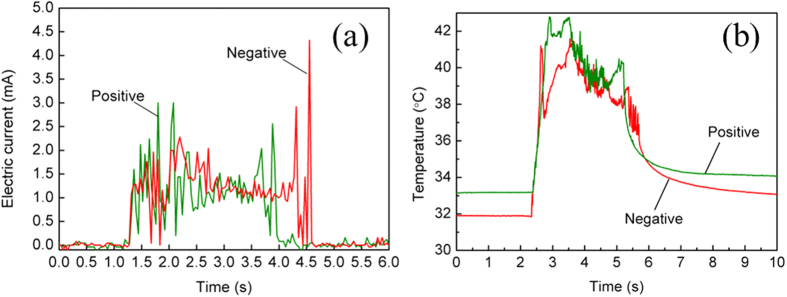
The variation curves of the electric currents (**a**) and the average temperatures (**b**) over time after discharge inception between two droplets under high voltages.

**Figure 4:**
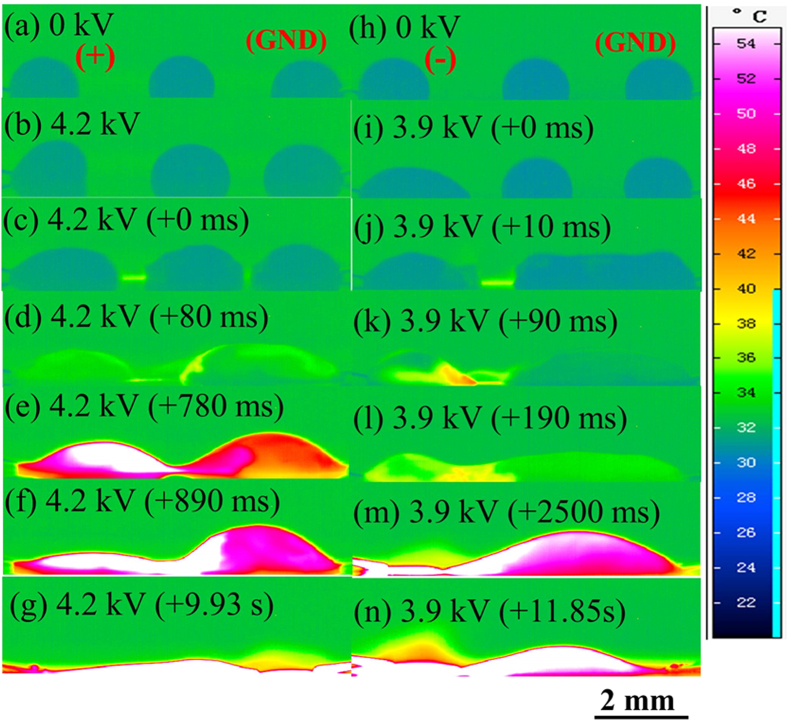
Side-view thermographic pictures of three aligned water droplets (the intermediate droplet was located at the middle between two extreme droplets) (droplet volume: 2 μL, the separation between two extreme droplets: 7 mm) on the SR surface under a high voltage (the left electrode was energized, and the right one grounded). (**a–g**): positive voltages; (**h–n**): negative voltages.

**Figure 5:**
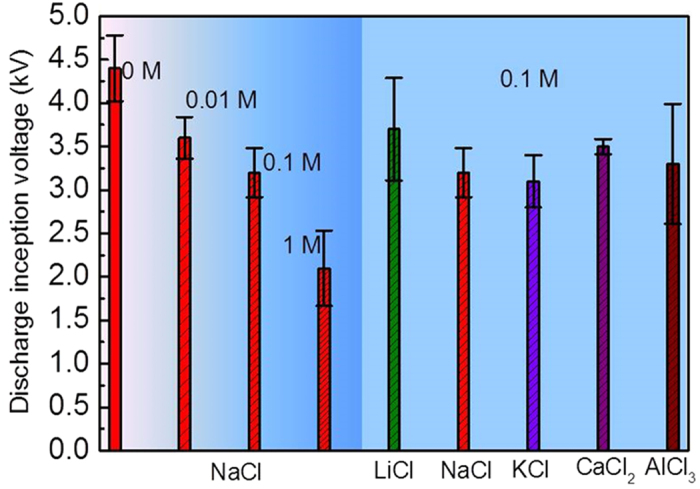
Discharge inception voltages of different salt droplets on the SR surface under a high positive voltage. (Droplet volume: 2 μL, the separation between two extreme droplets: 4 mm).

**Figure 6:**
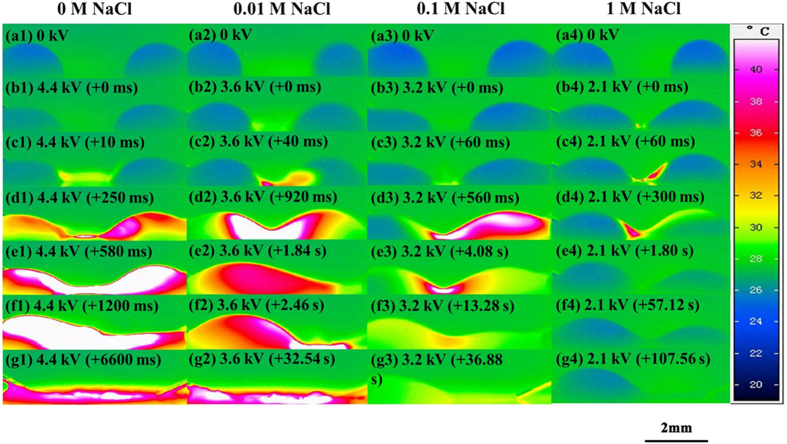
Side-view thermographic pictures of droplets of NaCl solutions with different concentrations (droplet volume: 2 μL, droplet separation: 4 mm) on the SR surface under a high positive voltage (the left electrode was energized, and the right one grounded).

**Figure 7:**
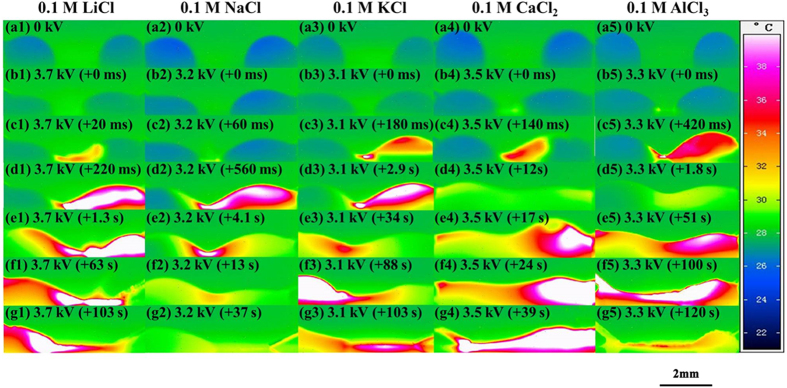
Side-view thermographic pictures of droplets of different salt (LiCl, NaCl, KCl, CaCl_2_ and AlCl_3_) solutions (droplet volume: 2 μL, droplet separation: 4 mm) on the SR surface under a high positive voltage (the left electrode was energized, and the right one grounded).

Most experiments were conducted with water droplets on flat horizontal insulator’s surfaces, and the test insulator’s materials were hydrophobic silicone rubber, SR and polytetrafluoroethylene, PTFE, which were typical insulating materials in high power applications. As shown in Fig. 1, metal electrodes, which consisted of copper wire (diameter, *d*_electrode_ = 0.2 mm) were dipped into the center point of the droplets, and the gap distance between the center points was defined as the droplet separation. DC voltages between the electrodes were applied and increased gradually until the inception of droplet deformation or discharge between droplets. The potential drop across the resistance (500 Ω) was fed directly to a digital storage to measure the discharge current.

### Preparation of droplets

Aqueous droplets having the same volume were placed on the top of the solid surface with a micropipette. Droplets of deionized water and various chloride salt solutions (sodium chloride, NaCl, lithium chloride, LiCl, potassium chloride, KCl, calcium chloride, CaCl_2_, aluminium chloride, AlCl_3_) were used. The electric conductivities of the salt solutions used in the present work are summarized in Table 1. The Young’s angles (*Θ*) of deionized water and salt solution droplets on the SR and PTFE surfaces were around 100°.

### Visualization and temperature measurement of droplets

The side views of the temperature variation and the movement of droplets were obtained by using an infrared thermographic camera with a microscopic lens (InfraTec GmbH, resolution: 10 μm/pixel, speed: 100 frames/s). Meanwhile, the top views of the droplet behavior were also obtained with an optical microscope and recorded by using a high-speed charged coupled device (CCD) camera at 400 frames/s. Thermodynamic equilibrium of the droplets between the three (solid/liquid/gas) phases was reached prior to applying a voltage, when the alterations of the droplets’ shape and the temperature became negligible. For calibration purpose, water with different temperatures were measured with the infrared thermocamera and a thermal meter, and a relationship between the real temperature *T*_r_ (°C) of water and the nominal value *T*_m_ (°C) measured with the infrared thermocamera was fitted, i.e., 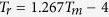. However, the nominal value was mostly discussed for convenience in the following parts.

## Additional Information

**How to cite this article**: Xie, G. *et al*. Sessile multidroplets and salt droplets under high tangential electric fields. *Sci. Rep*. **6**, 25002; doi: 10.1038/srep25002 (2016).

## Supplementary Material

Supplementary Information

## Figures and Tables

**Table 1:** Electric conductivities of the salt solutions used in the present work (20 °C).

Salt solution	NaCl 0 M (Deionized Water)	NaCl 0.01 M	NaCl 0.1 M	NaCl 1 M	LiCl 0.1 M	KCl 0.1 M	KCl 1 M	CaCl_2_ 0.1 M	AlCl_3_ 0.1 M
Electric conductivity (mS/cm)	~0.001	~0.1-~1	9.9	98.6	8.4	11.7	102.1	17.1	22.1
