# Supplementary material for: Sessile multidroplets and salt droplets under high tangential electric fields
Source: Sci Rep. 2016 Apr 28;6:25002. doi: 10.1038/srep25002 (PMC4848563; doi:10.1038/srep25002)
Supplement: Supplementary Information [file srep25002-s1.doc]

**Supplementary Information File**

**Sessile multidroplets and salt droplets under high tangential electric fields**

Guoxin Xie,^1^ Feng He, ^1^ Xiang Liu,^1^ Lina Si,^1,2^* Dan Guo,^1^*

*^1^State Key Laboratory of Tribology, Tsinghua University, Beijing 100084, China*

*^2^School of Mechanical Engineering, Beijing Institute of Technology, Beijing 100081, China*

Corresponding authors: * [silina_thu@163.com](mailto:silina_thu@163.com)(Lina); * [guodan26@tsinghua.edu.cn](mailto:guodan26@tsinghua.edu.cn) (Dan)


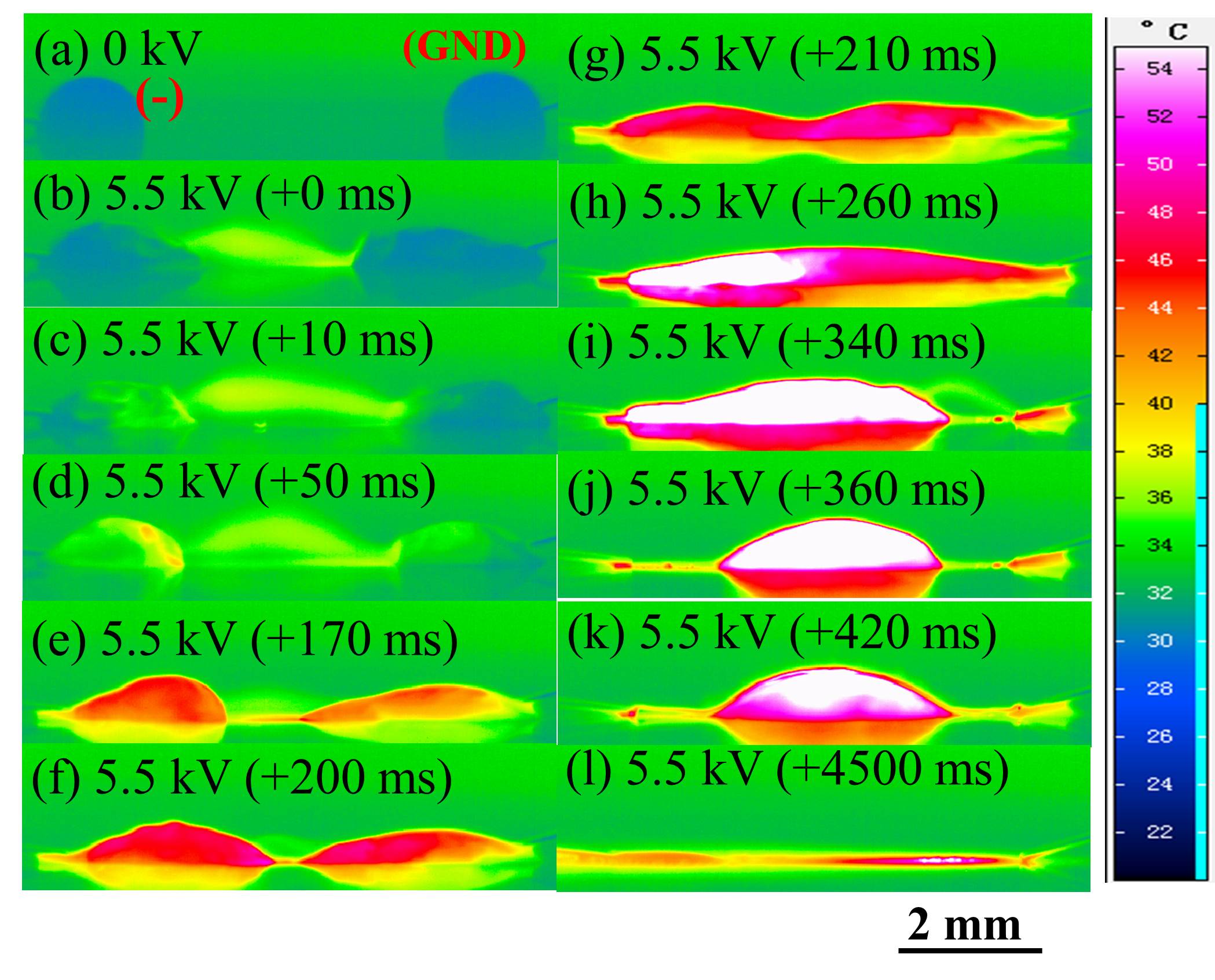


Figure S1. Side-view thermographic pictures of two deionized water droplets (droplet volume: 2 μL, droplet separation: 7 mm) on the SR surface under a high negative voltage (the left electrode was energized, and the right one grounded).

After the negative voltage was applied onto the energized electrode and increased to 5.5 kV, discharge started from the grounded droplet to the opposite droplet [Fig. S1(a)-(d)]. The direction of discharge path was just opposite to that under positive discharge in Fig. 2. Moreover, it could be seen that both the grounded droplet and the grounded droplet deformed equally obvious at the discharge inception instant, probably due to the increasing amount of ions in the grounded droplet once the discharge initiated. In the same way, the temperature of the energized droplet increased initially, from the right side of the droplet and gradually spread to the left side [Fig. S1(e) and (f)]. Afterwards, being similar to positive discharge in Fig. 2, water bridge formation [Fig. S1(g)], droplet coalescence [Fig. S1(h)], subsequent detachment of the merged droplet from the electrodes [Fig. S1(i)-(k)], intermittent discharge between the merged droplet and two electrodes, and finally drying out of the droplet took place sequentially[Fig. S1(l)].

In Fig. S2, the electric currents during the discharges between the three droplets were smaller (mostly ca. 0.5 mA) than those of the two droplet cases (ca. 1.5 mA) in Fig. 3(a). Moreover, both of the data in Fig. 3 and Fig. S2 suggested that negative discharge was longer than under positive discharge. In other words, positive discharge was more intensive, and the temperature changes correlated well with the changes of the electric currents.


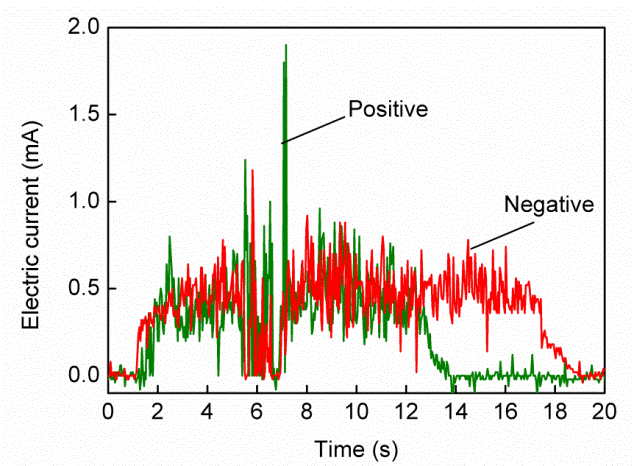

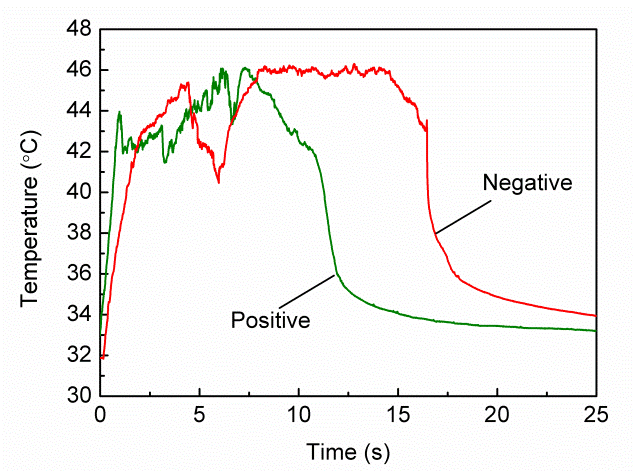


(a) (b)

Figure S2. The variation curves of the electric currents (a) and the average temperatures (b) over time after discharge inception between three aligned droplets under high voltages, corresponding to Fig. 4.


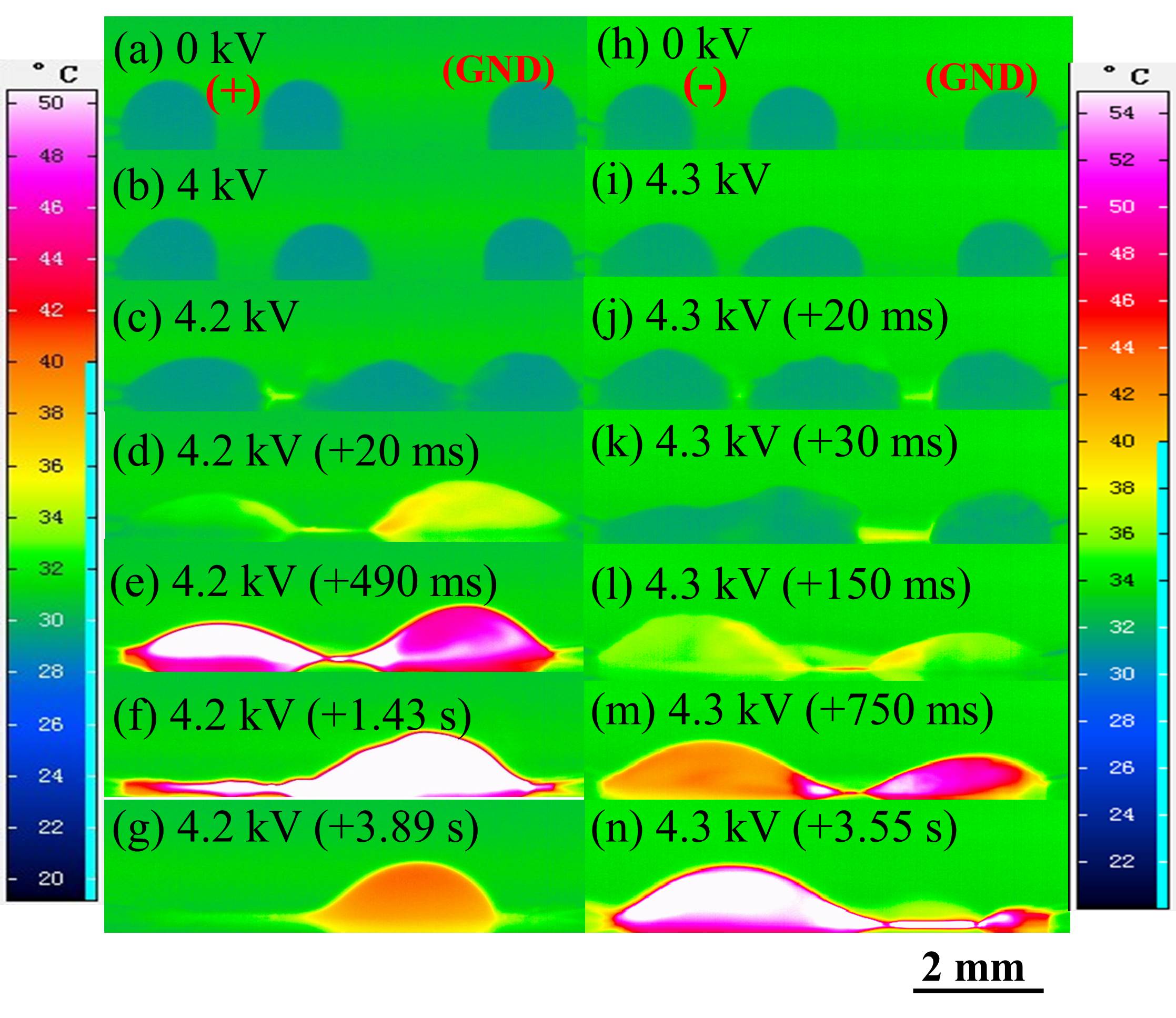


Figure S3. Side-view thermographic pictures of three aligned water droplets (the inserted droplet was located near the energized droplet) (droplet volume: 2 μL, the separation between two extreme droplets: 7 mm) under a high positive voltage (a)-(g) and negative voltage (h)-(n).


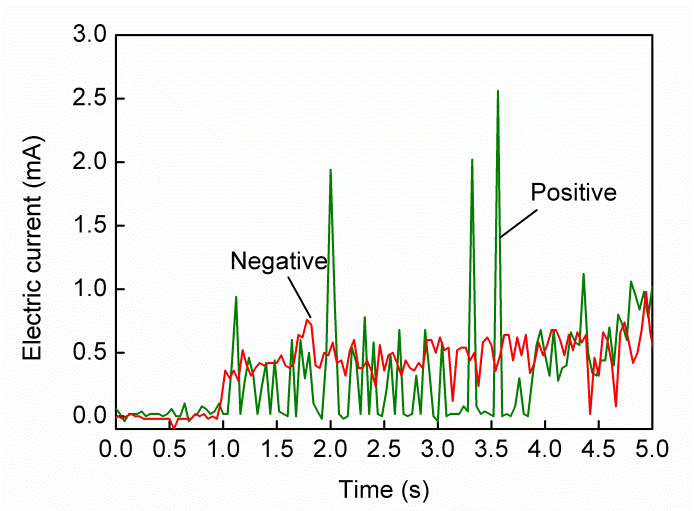


Figure S4. The variation curves of the electric currents over time after discharge inception between three aligned droplets under high voltages, corresponding to Fig. S3.

The position of the inserted droplet was changed to investigate its influence on the droplet coalescence and discharge activity. Thermographic pictures of three aligned water droplets with the inserted droplet near the energized droplet under high voltages are shown in Fig. S3. In the case of applying a positive voltage, even though the inserted droplet was near the energized droplet, droplet coalescence occurred firstly between the inserted droplet and the grounded droplet after discharge initiation [Fig. S3(a)-(g)]. The subsequent droplet interaction and the discharge activity between droplets were generally close to the case of the inserted droplet at the middle between two extreme droplets in Fig. 4. In contrast, when the energized droplet was negatively charged, the inserted droplet firstly coalesced with the energized droplet [Fig. S3(h)-(n)]. Then, the subsequent process was contrary to the case of positive discharge [Fig. S3(a)-(g)], in that the grounded droplet gradually drained to the larger energized droplet. The variation curves of the electric currents in Fig. S4 also suggested that positive discharge was more intensive with many burst pulses.


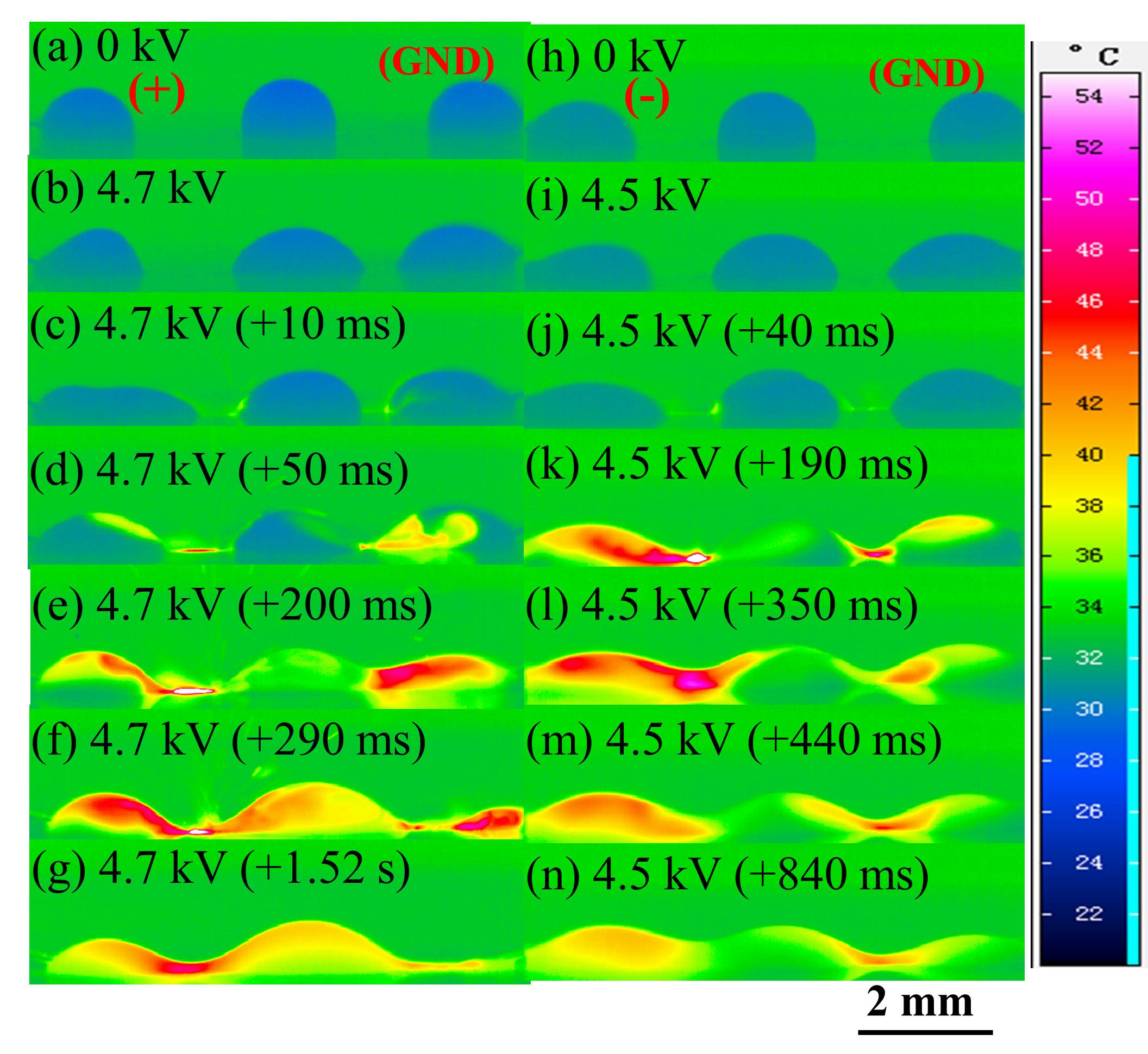


Figure S5. Side-view thermographic pictures of three aligned water droplets with the KCl droplet inserted at the middle between the two extreme droplets (droplet volume: 2 μL, the separation between two extreme droplets: 7 mm) on the SR surface under a high positive voltage (a)-(g) and negative voltage (h)-(n). The left electrode was energized, and the right one grounded).


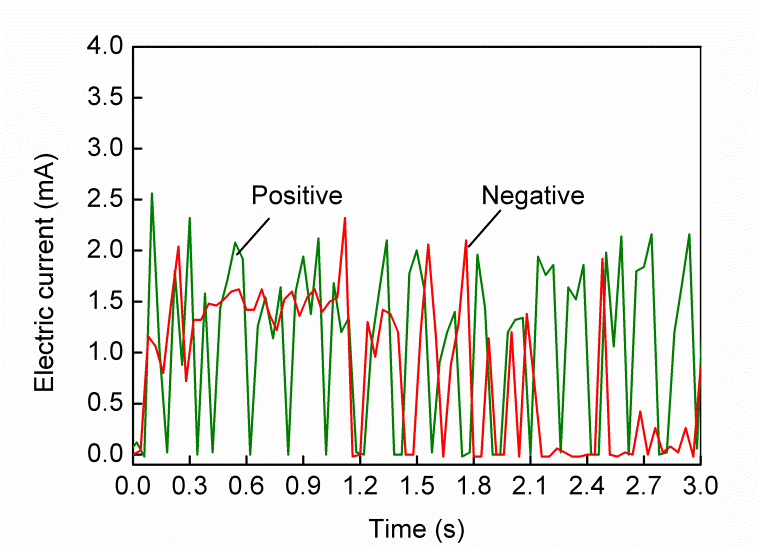

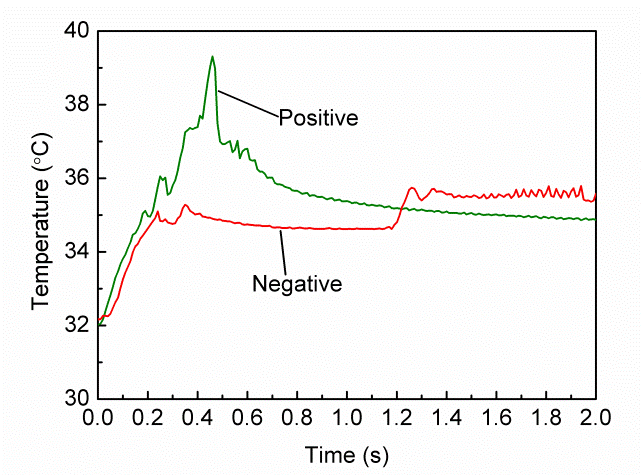


(a) (b)

Figure S6. The variation curves of the electric currents (a) and the average temperature (b) over time after discharge inception between three aligned droplets with the KCl droplet at the middle between the two extreme droplets under high voltages, corresponding to Fig. S5.

In the next part, a KCl droplet was placed as the inserted droplet, and the energized and grounded droplets were of deionized water. Thermographic pictures of these droplets under high negative voltages are shown in Fig. S5(a)-(g) (positive) and Fig. S5(h)-(n) (negative), and the inserted droplet was located at the middle between two extreme droplets. The variations of the corresponding electric currents and the average temperatures after discharge inception are shown in Fig. S6. In the case of applying the positive voltage, the droplet deformation at the energized side was more obvious [Fig. S5(a) and (b)]. After discharge inception, the discharge path could be observed from the energized droplet, via the inserted droplet and to the grounded droplet, as shown in Fig. S5(c) and (d). Afterwards, droplet coalescence did not occur as fast as the case where the inserted droplet was of deionized water in Fig. 4. The temperature rise of the grounded droplet was the highest, and that of the energized droplet was also obvious, while the temperature of the inserted droplet increased relatively slowly [Fig. S5 (e)]. It can be seen from Fig. S5I (f) that the grounded droplet was drained into the inserted droplet with a very thin liquid film connecting the grounded electrode with the middle droplet. In Fig. S5I (g), the remaining droplet almost remained with a liquid bridge of a slightly higher temperature between them. In the case of applying a negative voltage, as shown in Fig. S5(h)-(n), the direction of the initial discharge path was opposite to the case with a positive voltage, i.e., from the grounded droplet via the inserted one to the energized one. Although asymmetric temperature distribution of the two extreme droplets could be still observed, the temperature rises of these droplets were not as high as the case applying a positive voltage, as also demonstrated in Fig. S6 where the lower electric current and less temperature rise for the negative case can be seen, resulting in the absence of the drainage of the extreme droplets to the inserted one.


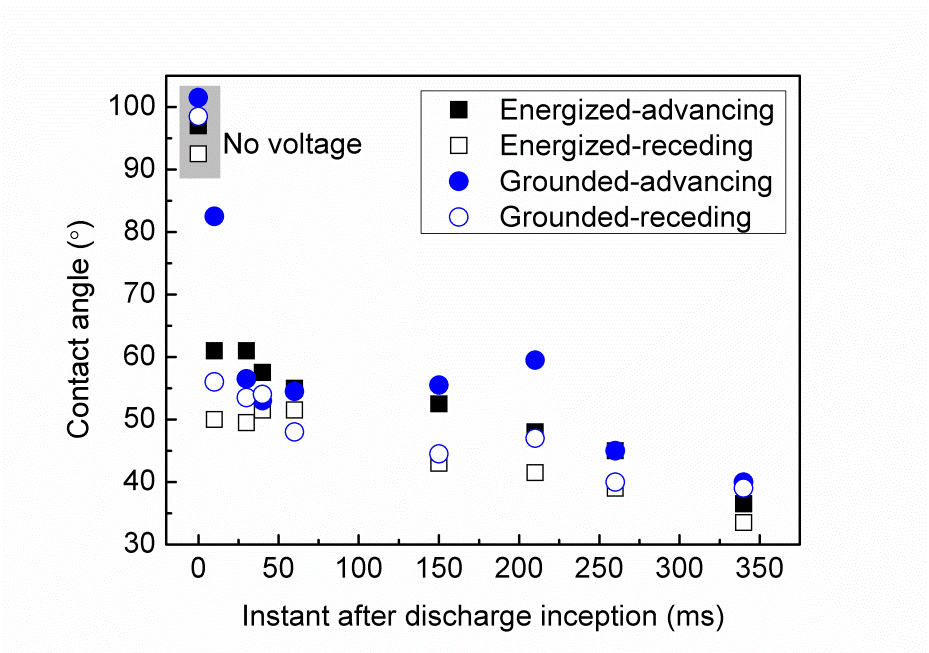


Figure S7. The variations of the contact angles (advancing and receding) over time after discharge inception between two water droplets under positive high voltages, corresponding to Fig. 2 (a) and (c)-(g). It should be noted that some of the figures at different time instants were not shown in Fig. 2.


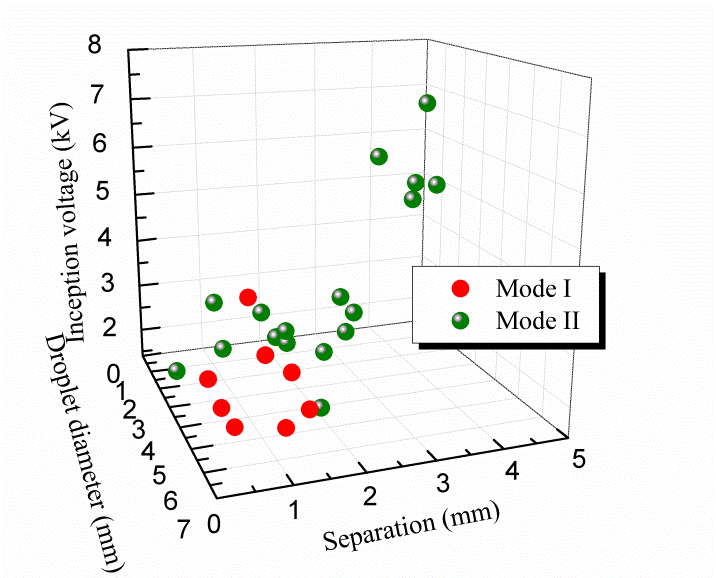


Figure S8. The inception voltages of discharge or droplet coalescence for the two-droplet configuration on the PTFE surface with different droplet diameters and separations. Positive voltage was applied onto the energized electrode.

Figure S8 shows the relationships between the inception voltages of discharge or droplet coalescence for the two-droplet configuration on the PTFE surface and the droplet diameter and separation. Mode I was defined as the state that no visible discharge occurred before droplet coalescence, i.e., droplet coalescence took place firstly after a high voltage was applied due to droplet deformation/elongation. Mode II was defined as the state that obvious discharge could be observed before the occurrences of droplet elongation and coalescence. When the droplet size was small or the droplet separation was large, the droplet behavior after applying a high voltage was in Mode II, and otherwise in Mode I.
